# Death anxiety in the time of COVID-19: theoretical explanations and clinical implications

**DOI:** 10.1017/S1754470X20000215

**Published:** 2020-06-11

**Authors:** Rachel E. Menzies, Ross G. Menzies

**Affiliations:** 1 School of Psychology, University of Sydney, Sydney, Australia; 2 Graduate School of Health, University of Technology Sydney, Sydney, Australia

**Keywords:** COVID-19, death anxiety, existential, transdiagnostic

## Abstract

The recent COVID-19 pandemic has triggered a surge in anxiety across the globe. Much of the public’s behavioural and emotional response to the virus can be understood through the framework of terror management theory, which proposes that fear of death drives much of human behaviour. In the context of the current pandemic, death anxiety, a recently proposed transdiagnostic construct, appears especially relevant. Fear of death has recently been shown to predict not only anxiety related to COVID-19, but also to play a causal role in various mental health conditions. Given this, it is argued that treatment programmes in mental health may need to broaden their focus to directly target the dread of death. Notably, cognitive behavioural therapy (CBT) has been shown to produce significant reductions in death anxiety. As such, it is possible that complementing current treatments with specific CBT techniques addressing fears of death may ensure enhanced long-term symptom reduction. Further research is essential in order to examine whether treating death anxiety will indeed improve long-term outcomes, and prevent the emergence of future disorders in vulnerable populations.

**Key learning aims:**

(1) To understand terror management theory and its theoretical explanation of death anxiety in the context of COVID-19.
(2) To understand the transdiagnostic role of death anxiety in mental health disorders.
(3) To understand current treatment approaches for directly targeting death anxiety, and the importance of doing so to improve long-term treatment outcomes.

## Introduction

In December 2019, a novel coronavirus was first detected in the city of Wuhan, China. Within five weeks, the virus, now named COVID-19, began to dominate global headlines. By mid-May 2020, COVID-19 had resulted in the deaths of more than 300,000 people worldwide, with nearly 4.5 million cases confirmed (World Health Organization, 2020). As cases increased, governments around the world began closing borders, and introducing social distancing restrictions and lockdown orders, in an effort to slow the rapid acceleration of the virus. Prior to many of these government responses, reports emerged of individuals choosing to self-isolate, as mass panic swept through communities in waves. Anecdotal reports of verbal and physical aggression in grocery stores, hoarding of antibacterial products and other supplies, and racist abuse of individuals with Asian appearance increased as fear took over across the world (Devakumar *et al.*, 2020; Garfin *et al*., 2020). As individuals scrambled to prevent the threat of COVID-19 in any way they could, online sales of ‘immune boosters’ and untrialled medicines increased. Analyses of Google data across just 14 days in March 2020 revealed a total of 216,000 searches for where to purchase chloroquine and hydroxychloroquine, two drugs which were touted by the media as potentially effective, despite the existing clinical evidence for the efficacy of these drugs being inconclusive (Liu *et al*., 2020). Emerging research data are already revealing high levels of anxiety concerning the virus, with findings from nearly 5000 participants suggesting that greater perceived severity of the virus is associated with poorer mental health outcomes (Li *et al*., 2020).

Arguably, this response from the public should not come as a surprise. Fears of death have been proposed to be a central and universal part of the experience of being human (Becker, 1973). In fact, there is evidence of humans grappling with death anxiety for as long as our species has been recording its history (Menzies, 2018b). We are the only species that we know of that has the cognitive capacity to contemplate and anticipate our own death, yet this impressive ability comes with a downside; we are destined to live our lives ‘forever shadowed by the knowledge that we will grow, blossom, and inevitably, diminish and die’ (Yalom, 2008, p. 1). On the one hand, people may develop adaptive ways of coping with their fear of death, such as building meaningful relationships and leaving a positive legacy (Yalom, 2008). On the other hand, awareness of death may also produce a powerful sense of fear or meaninglessness, and may drive a number of maladaptive coping behaviours (Menzies, 2012). Some of these behaviours (e.g. avoidance) may underlie numerous mental health conditions, while other behaviours may appear, on the surface, not directly linked to death at all. How might our fears of death be shaping our everyday behaviour in ways that we are not even aware of?

## Terror management theory

Terror management theory (TMT), a social psychological theory based on the work of cultural anthropologist Ernest Becker, is the leading psychological framework for explaining this effect of death fears on human behaviour (Greenberg *et al*., 1992). TMT posits that our awareness of our own death produces a crippling terror, and that humans have developed two distinct buffers in order to allay this fear: cultural worldviews, and self-esteem. Cultural worldviews involve shared symbolic concepts of the world, including identifying with cultural values or endorsing belief systems, such as the belief in an afterlife. Sharing these cultural worldviews is thought to offer a sense of ‘symbolic immortality’, by giving an individual a sense of permanence and meaning in the face of death. Secondly, self-esteem, gained through fulfilling the expectations of our cultural worldview, is also said to buffer death anxiety, by making one feel like a valuable member of their culture, who will be remembered after death (Greenberg, 2012).

TMT also proposes that humans use different defence mechanisms depending on whether thoughts of death are within or outside of conscious awareness. According to this ‘dual process model’, when thoughts of death are conscious, we engage in ‘proximal defences’, which include suppressing these thoughts (e.g. turning off a news report about COVID-19 death tolls), denying one’s vulnerability (e.g. ‘I’m not in a high risk group, so I’ll probably be fine’), or trying to prevent death (e.g. cleaning down all home surfaces with antibacterial wipes) (Pyszczynski *et al*., 1999). On the other hand, when thoughts of death leave conscious awareness, we instead engage in ‘distal defences’, which involve bolstering our two buffers (e.g. by endorsing our cultural worldviews, or enhancing our self-esteem).

Findings from hundreds of studies have demonstrated support for TMT (Burke *et al*., 2010). Primarily, these studies have involved a ‘mortality salience’ design, in which participants in one condition are reminded of their mortality, while participants in the control condition are reminded of an aversive topic that is unrelated to death. These studies have shown that reminders of death drive a vast array of human behaviours, including intention to purchase products (Dar-Nimrod, 2012), driving behaviour (Taubman-Ben-Ari *et al*., 1999), and even suntanning (Routledge *et al*., 2004). Despite some recent studies questioning the replicability of TMT results (e.g. Klein *et al*., 2019), follow-up studies have demonstrated that classic TMT findings do replicate when sufficiently powered (Chatard *et al*., 2020). In addition, Burke *et al*.’s (2010) review of 277 TMT experiments found that death reminders yielded moderate effects on a range of behavioural variables, with little evidence of publication bias, further highlighting the strength of mortality salience effects. Given this, what role might death anxiety be playing in the current pandemic?

## Death anxiety and the COVID-19 pandemic

With the exception of a handful of studies, the majority of TMT research has been conducted under laboratory conditions; i.e. for those in the mortality salience condition, death is usually primed in the form of two short questions about one’s death, which participants are asked to respond to. COVID-19 offers an unusual scenario, in which mortality is made salient nearly constantly, given the daily updates on death tolls from the news and social media, and ubiquitous visible death cues in the form of face masks, anti-bacterial sprays and wipes, social distancing and public health campaigns. Supporting this idea, laboratory findings have demonstrated that reflecting on current epidemics or virus outbreaks (e.g. Ebola, swine flu) produces comparable findings to standard mortality salience primes, increasing the accessibility of death-related thoughts, and increasing defensive behaviour (e.g. Arrowood *et al*., 2017; Bélanger *et al*., 2013; Van Tongeren *et al*., 2016).

Although it is currently unknown what the long-term effects of mortality salience primes are, the consequence on human behaviour of even minor, subtle reminders of death under laboratory conditions have much to tell us about the behaviours observed during the current pandemic. First, from this perspective, the observed reports of both covert and overt racism towards Asian individuals are unsurprising. These observations are supported by a recent study that found a positive relationship between coronavirus-related anxiety and avoidance of Chinese food and products (Lee, 2020), echoing similar observations of avoidance of Chinese people following the 2003 SARS outbreak (Keil and Ali, 2006).

These experiences offer a real-world confirmation of the TMT laboratory findings that reminders of death lead people to feel more hostile towards those of different cultural backgrounds to their own, as they are seen as a threat to one’s own worldviews. Findings across a number of studies reveal that reminders of death increase stereotypical thinking about people of other races (Schimel *et al*., 1999), increase aggression against those who criticise one’s nation (McGregor *et al*., 1998), and lead White participants to hold more favourable reactions to White pride advocates (Greenberg *et al*., 2001). One study even found that Germans interviewed in front of a cemetery reported strongly preferring German products over foreign products, whereas Germans interviewed in front of a shop did not show this preference (Jonas *et al*., 2005). Similar effects have been observed in more than 12 countries worldwide (Greenberg and Kosloff, 2008). So, much of the recent upsurge in xenophobia, or even hostility towards those with different political views, can be explained by the TMT notion that bolstering our cultural worldviews, and aggressing against those that threaten them, are one means of gaining a sense of symbolic immortality. This idea is further supported by the recent observation of mutual discrimination between East Asian societies in the midst of the pandemic (e.g. individuals in Taiwan avoiding contact with Koreans and Japanese individuals; Lin, 2020).

Whilst the bolstering of one’s cultural worldviews is an example of distal defences being engaged during the pandemic, proximal defences, in the form of attempts to ward off death (e.g. spikes in purchases of hydroxychloroquine, a drug falsely touted as a cure to the virus) or denial have also been observed (Jong-Fast, 2020). Furthermore, despite the unsurprising recency of much of the research, some preliminary data support the idea that death anxiety may be driving a significant amount of psychological distress during this pandemic. Evaluation of the psychometric properties of the Fear of COVID-19 Scale revealed that the item ‘I am afraid of losing my life because of coronavirus-19’ had the highest factor loading, suggesting that one’s worry about one’s own fatality risk is highly predictive of broad fears of the virus (Ahorsu *et al*., 2020). Data from 1210 residents of China revealed that estimates of fatality also appear to specifically predict their psychological distress, with low estimates of one’s own survival from COVID-19 predicting greater levels of stress and depression on the Depression, Anxiety and Stress Scale DASS-21 (Wang *et al*., 2020).

One large study of 810 Australians specifically explored fears of death in the context of the pandemic (Newton-John *et al*., 2020). The findings revealed a significant positive correlation between death anxiety and anxious beliefs and behaviours related to COVID-19 (e.g. estimated likelihood of contracting the virus, estimated likelihood of wearing a mask in public, etc.), in addition to self-reported health anxiety, and overall psychological distress. Furthermore, participant responses to items assessing beliefs surrounding the virus indicated a heightened perception of threat. For example, when participants were asked how likely they would be to die if they contracted COVID-19 in the next 18 months, the mean likelihood estimate was 22%, a figure more than 11 times the actual Australian case fatality rate of <2%.

So, while death anxiety may indeed be a driving factor in everyday human behaviour, it appears more relevant than ever in the context of the current pandemic. COVID-19 may be understood as a real-life and ever-present mortality salience prime, influencing people’s behaviour in ways they may not even be consciously aware of. Early findings suggest that fears of death predict anxiety about the virus, which in turn is shown to predict broader psychological distress. These findings may suggest a causal relationship between death anxiety and psychological distress, and this relationship may be exacerbated in the current pandemic.

## Death anxiety and psychopathology

Death anxiety has been proposed to be a transdiagnostic construct, underpinning a range of different mental health conditions (Iverach *et al*., 2014). For instance, fears of death may manifest in the frequent reassurance seeking from doctors, checking of one’s body, and requests for medical testing seen in the somatic symptom-related disorders (Furer *et al*., 2007). In a similar vein, panic disorder often features worries about heart attacks during panic attacks, in addition to repeated appointments with cardiac specialists to allay these concerns (Starcevic, 2007). Specific phobias have been argued to have death anxiety at their core for over a century (Kingman, 1928), with all of the common phobic objects having the potential to directly result in death (e.g. fears of spiders, snakes, flying and heights). Fears of death have also been argued to play a central role in various presentations of obsessive compulsive disorder, as clients attempt to prevent death by illness (in the contamination subtype), household fire or electrocution (in compulsive checking), and death to oneself or another due to acting on intrusive thoughts (as seen in aggressive obsessions) (Menzies and Dar-Nimrod, 2017; Menzies *et al*., 2015). Existential concerns have also been argued to play a role in the depressive disorders, with concerns surrounding death and meaninglessness being a common theme (Ghaemi, 2007; Simon *et al*., 1998).

A number of studies have demonstrated significant relationships between self-reported death anxiety and symptomology of various disorders, including separation anxiety (Caras, 1995), hypochondriasis (Noyes *et al*., 2002), post-traumatic stress disorder (Martz, 2004), depression (Ongider and Eyuboglu, 2013) and eating disorders (Le Marne and Harris, 2016). Results from one large clinical sample found significant and positive correlations between death anxiety and number of lifetime mental health diagnoses, number of medications for mental health, DASS-21 depression, anxiety and stress scores, as well as the symptom severity of 12 different disorders (Menzies *et al*., 2019). Notably, these relationships remained significant after controlling for neuroticism, suggesting the unique role of death anxiety in psychopathology.

While limited conclusions regarding causality can be drawn from such correlational designs, a handful of studies have explored the causal role of death anxiety in mental illnesses using a mortality salience design. These have revealed that reminders of death increase avoidance of spider-related stimuli among spider phobics (Strachan *et al*., 2007), social avoidance (Strachan *et al*., 2007) and attentional biases towards threat among the socially anxious (Finch *et al*., 2016), and even restricted consumption of high caloric foods amongst women, suggesting the relevance of death anxiety in eating disorders (Goldenberg *et al*., 2005). While few studies have used clinical samples, one study investigated the effect of mortality salience on compulsive handwashing, utilising a large sample of treatment-seeking individuals diagnosed with OCD (Menzies and Dar-Nimrod, 2017). Participants were first primed with either death or a control topic. Following a short delay to allow the effects of the prime to become unconscious, they were asked to wash their hands. The findings revealed that reminders of death doubled the time spent handwashing. Notably, this increase in handwashing occurred despite no difference in reported anxiety or perceptions of cleanliness. Results from another recent mortality salience design appear particularly relevant to the current pandemic. Across a sample of participants with panic disorder or a somatic symptom-related disorder, reminders of death were shown to increase time spent checking one’s body for physical symptoms, increase perceived threat of one’s symptoms, and also increase intention to visit a medical specialist in the near future (Menzies *et al*., 2020). These findings suggest that death anxiety drives relevant anxious behaviour for those vulnerable to health-related worries.

Results from numerous studies appear to suggest that fear of death is indeed a transdiagnostic construct driving a number of mental health conditions, although further research using treatment-seeking and clinical samples is clearly warranted. If death anxiety does underlie numerous disorders, this may explain the ‘revolving door’ phenomenon often observed in clinical practice, in which an individual receives apparently successful treatment for one disorder, only to present with a distinctly different disorder at a later time point (Iverach *et al*., 2014, p. 590). If death anxiety is indeed ‘the worm at the core’ (James, 1985, p. 119) of the human psyche, then failing to treat it may result in individuals continuing to present with different mental health conditions at various points across their lifespan. Fear of death may need to be assessed and explicitly targeted in treatment in order to achieve long-term amelioration in symptoms and foster ongoing client wellbeing.

## Assessing death anxiety

As with any target of clinical treatments, a thorough assessment paves the way to the most effective treatments of death anxiety, tailored to the individual’s unique needs. The clinical interview in early sessions should focus on exploring the topic of death, including assessing for any early losses, memories, or experiences associated with death (Menzies and Veale, 2020). It is also essential to assess the individual’s specific worries or thoughts about death, as these can vary largely between individuals. For example, worries may revolve around the dying process itself (e.g. pain or loss of cognitive capacities), the feared death of a loved one, fears concerning eternal punishment in the afterlife, uncertainty surrounding life after death, or non-existence itself, and each theme may need to be addressed using distinctly different lines of cognitive challenging. Maladaptive behaviours the individual engages in should also be identified during the assessment stage, including any avoidance behaviours (e.g. avoiding the news, hospitals, flying or driving, or suppressing thoughts around death), reassurance seeking (e.g. from family or one’s doctor), self-medicating, or compensatory behaviours (e.g. excessive exercise) (Menzies and Veale, 2020). In the context of the current pandemic, it would be important to distinguish behaviours which are adaptive (i.e. behaviours generally recommended by health professionals and public officials, such as wearing a face mask when leaving the house, self-isolating when symptomatic, and regularly washing one’s hands for recommended durations) compared with those that are maladaptive (i.e. behaviours that are not in line with standard recommendations and disrupt the individual’s life, such as washing one’s hands for hours each day, or requesting repeated medical tests for the virus despite lack of symptoms).

Alongside a standard clinical interview, questionnaires can prove useful in measuring severity of death fears, as well as tracking change following treatment. One recent systematic review of death anxiety measures revealed that there is a strong need for rigorous measures which have been validated in clinical samples, and that many measures in this field lack adequate psychometric properties (Zuccala et al., 2019). Despite this, a number of measures may prove particularly useful in assessing death anxiety. These include the Collett-Lester Fear of Death Scale-Revised (Lester, 1990), which has been demonstrated to be responsive to treatment effects, and thus appears to be the best choice for exploring clinical change, and the Multidimensional Fear of Death Scale (Hoelter, 1979), for which means for various clinical groups have been reported (Menzies *et al*., 2019). The Death Attitude Profile-Revised (Wong *et al*., 1994) may also offer clinical utility, due to its unique assessment of adaptive attitudes, such as three distinct types of death acceptance, which have been shown to predict more positive outcomes (Tomer and Eliason, 2000).

## Treatment approaches

Despite being understood to be an ‘existential given’ (Yalom, 1980), empirical findings fortunately indicate that death anxiety can indeed be ameliorated. One recent meta-analysis examined the effects of randomised controlled trials on death anxiety (Menzies *et al*., 2018). This revealed that psychosocial interventions produced significant reductions in death anxiety relative to control conditions. Notably, this effect was found to be driven by cognitive behaviour therapy (CBT) interventions, which produced significantly greater improvements in death fears compared with other treatment modalities. In particular, CBT treatments centring on graded exposure therapy were found to be most effective. In fact, alternative treatment options examined by the meta-analysis failed to produce any significant change in death anxiety scores (Menzies *et al*., 2018). Given these meta-analytic findings, CBT appears to be the most appropriate treatment for addressing death anxiety, and various techniques for doing so have been proposed (see further, Menzies, 2018a; Menzies and Veale, 2020).

## Exposure therapy

A number of exposure therapy tasks have been recommended in order to ameliorate death anxiety. Of course, as with any exposure tasks, these should be specifically tailored to the individual’s own unique pattern of avoidance, and situations or themes that the individual has systematically avoided should be prioritised. One exposure task that can be tailored to the individual’s specific concerns is that of an ‘illness story’, recommended by Furer *et al*. (2007). This involves writing a vivid description of the death of oneself or a loved one, starting with the events leading up to the death (e.g. the initial diagnosis of a terminal illness), progressing to the death itself, followed by the imagined funeral and aftermath. A similar task is popularised by acceptance and commitment therapy, which involves vividly imagining one’s own funeral, and writing one’s own eulogy and a tombstone inscription (Hayes and Smith, 2005).

Other exposure tasks may involve visiting places associated with death that the client has avoided, such as hospitals, nursing homes, cemeteries or funeral homes. Reading obituaries online or in the newspaper may also offer valuable exposure opportunities, and clients should be encouraged to deliberately seek out those who have died around their own age (Furer *et al*., 2007). Preparing one’s will, or having discussions regarding end-of-life preferences, may also be considered as exposure tasks, and may serve the additional benefit of increasing the individual’s sense of control over their death (Furer *et al*., 2007; Henderson, 1990). Books (e.g. *When Breath Becomes Air* by Paul Kalanithi), films (e.g. *Blade Runner*, *Up*), television shows (e.g. *After Life*) and music (e.g. *All Things Must Pass* by George Harrison) related to death may all offer valuable and powerful opportunities for exposure, in addition to helping to normalise death.

## Cognitive approaches

Two thousand years ago, the Stoic philosophers of ancient Greece observed that ‘it is not things themselves that trouble people, but their opinions about things’ (Epictetus, 2018, p. 11). This principle lies at the heart of both Stoic philosophy (which emphasised the need to accept death as a universal event outside of our control) and CBT. All of us hold an array of beliefs surrounding death, which may fluctuate between being adaptive (e.g. the belief that we would ultimately cope with the death of a loved one) or maladaptive (e.g. the belief that dying will inherently involve pain and suffering). Beliefs of this latter type will understandably cause distress for many individuals, and should be explicitly identified and challenged in therapy. For example, in the context of COVID-19, the distress of some individuals will be grounded on over-estimating the probability of death from the virus; over-estimates of the fatality risk are commonplace (Newton-John *et al*., 2020). However, while standard treatments for anxiety may often involve disproving the client’s probability estimates (Kirk and Rouf, 2004), this is not recommended in treatments targeting death anxiety. Disproving the individual’s estimate of dying from any one particular cause (e.g. falling to one’s death, dying in a plane crash, or succumbing to COVID-19) only serves to address the proximal threat, and will probably do little to address their fear of their own inevitable death, from one cause or another. As such, it is central instead to focus on addressing the *cost* of death, rather than merely the probability. Clients should be guided to cultivating an attitude of ‘neutral acceptance’ towards death; that is, an acceptance of death as a universal fact outside of one’s control, and therefore neither good nor bad (Wong *et al*., 1994). Standard cognitive challenging techniques can also be used to challenge unrealistic beliefs surrounding death. For example, for individuals fearing pain associated with dying, corrective information may be provided in the form of information from palliative care, and research indicating that dying is less unpleasant than people typically imagine.

Notably, theoretical orientations outside of standard CBT may also prove valuable in shifting clients’ attitudes towards death. Approaches from existential psychotherapy may be particularly relevant, and Yalom (1980) outlines many relevant treatment recommendations from an existential lens. For example, for clients who express anxiety surrounding the concept of non-existence, Yalom (2008) recommends the use of the Stoic ‘symmetry’ argument, which proposes that humans have already experienced non-existence, that is, prior to their birth. That is, death ‘returns us to that peace in which we reposed before we were born. If someone pities the dead, let him also pity those not yet born’ (Seneca, 2018). These clients may also be encouraged to foster gratitude for ever coming into existence at all, an idea persuasively expressed by Richard Dawkins, who notes that ‘We are going to die, and that makes us the lucky ones’, as we have ‘won the lottery of birth against all odds’ (Dawkins, 1998, p. 1). In order to help build identification with this idea, one exercise may involve estimating the likelihood of one’s existence, by calculating the probability of one’s parents ever meeting, followed by grandparents, and so forth (Menzies, 2012), in order to help the client focus on the incredible unlikelihood of their own DNA sequence ever existing at all, rather than focusing on the tragedy of their own impermanence.

## Conclusion

The recent COVID-19 pandemic has caused an understandable surge in anxiety across the globe. Much of the behavioural response to COVID-19 can be understood through the lens of terror management theory, which argues that death anxiety drives much of human behaviour (Greenberg, 2012). From this perspective, reminders of death (of which there are many in the current pandemic), produce increases in attempts to avoid a physical death (such as by wearing protective gear or self-isolating) or ensure a symbolic immortality (such as by bolstering one’s cultural worldviews, and aggressing against those that threaten them). Death anxiety, which has recently been proposed to be a transdiagnostic construct (Iverach *et al*., 2014), appears to be more relevant now than ever before. In addition to predicting anxiety related to COVID-19 (Newton-John *et al*., 2020), fear of death has also been shown to play a causal role across a number of mental health conditions (Menzies and Dar-Nimrod, 2017; Menzies *et al*., 2020; Strachan *et al*., 2007).

Given this, current standard treatments for mental health conditions may benefit from addressing death anxiety directly, in order to prevent the ‘revolving door’ often seen in mental health services (Iverach *et al*., 2014, p. 590). Fortunately, CBT has been demonstrated to produce significant reductions in death anxiety, with exposure appearing to be particularly effective (Menzies *et al*., 2018). Complementing current treatments with specific CBT techniques addressing fears of death may help to ensure the best long-term outcomes for clients, and protect the individual from future disorders. However, further research is needed to examine whether treating death anxiety will in fact reduce the likelihood of future mental health problems.

## Key practice points

(1) Increasing evidence suggests that death anxiety is a key transdiagnostic construct, and may contribute to various mental health conditions.
(2) Standard treatments for a variety of disorders may need to be supplemented with specific treatment targeting death anxiety.
(3) Recent evidence demonstrates that death anxiety can be effectively reduced using CBT with a focus on exposure therapy.
(4) We suggest a number of CBT treatment strategies, including cognitive reframing of unhelpful thoughts, and exposure tasks tailored to the feared situations, themes or images the individual avoids.
(5) Future research is needed to examine whether directly addressing death anxiety does indeed produce long-term improvement in symptoms, and prevent future disorders.
